# Novel insights into the mechanism of SepL‐mediated control of effector secretion in enteropathogenic *Escherichia coli*

**DOI:** 10.1002/mbo3.571

**Published:** 2017-12-26

**Authors:** Meztlli O. Gaytán, Julia Monjarás Feria, Eduardo Soto, Norma Espinosa, Julia M. Benítez, Dimitris Georgellis, Bertha González‐Pedrajo

**Affiliations:** ^1^ Departamento de Genética Molecular Instituto de Fisiología Celular Universidad Nacional Autónoma de México Ciudad de México Mexico

**Keywords:** EPEC, EscV, injectisome, secretion hierarchy, SepL, T3SS

## Abstract

Type three secretion systems (T3SSs) are virulence determinants employed by several pathogenic bacteria as molecular syringes to inject effector proteins into host cells. Diarrhea‐producing enteropathogenic *Escherichia coli* (EPEC) uses a T3SS to colonize the intestinal tract. T3S is a highly coordinated process that ensures hierarchical delivery of three classes of substrates: early (inner rod and needle subunits), middle (translocators), and late (effectors). Translocation of effectors is triggered upon host‐cell contact in response to different environmental cues, such as calcium levels. The T3S substrate specificity switch from middle to late substrates in EPEC is regulated by the SepL and SepD proteins, which interact with each other and form a trimeric complex with the chaperone CesL. In this study, we investigated the link between calcium concentration and secretion regulation by the gatekeeper SepL. We found that calcium depletion promotes late substrate secretion in a translocon‐independent manner. Furthermore, the stability, formation, and subcellular localization of the SepL/SepD/CesL regulatory complex were not affected by the absence of calcium. In addition, we demonstrate that SepL interacts in a calcium‐independent manner with the major export gate component EscV, which in turn interacts with both middle and late secretion substrates, providing a docking site for T3S. These results suggest that EscV serves as a binding platform for both the SepL regulatory protein and secreted substrates during the ordered assembly of the T3SS.

## 1 INTRODUCTION

Type III secretion systems (T3SSs) are essential virulence determinants of many animal and plant bacterial pathogens, as they serve to inject virulence proteins, or effectors, directly into the host cell cytoplasm in a process called translocation (Buttner, 2012; Gaytan, Martinez‐Santos, Soto, & Gonzalez‐Pedrajo, 2016). These effectors modify host cell physiology for the benefit of the bacteria (Dean, 2011; Tosi, Pflug, Discola, Neves, & Dessen, 2013). T3SSs or injectisomes are employed by pathogenic bacteria that threaten human health, including enteropathogenic *Escherichia coli* (EPEC), which colonizes the small intestine and produces a singular histopathological modification called the attaching and effacing (A/E) lesion. This alteration is characterized by the effacement of epithelial microvilli, the intimate attachment of the bacteria to the host cell, and finally the development of an actin‐rich pedestal‐like structure beneath the adherence site (Kaper, Nataro, & Mobley, 2004). The effectors required for the formation of the A/E lesion are encoded in a pathogenicity island known as locus of enterocyte effacement (LEE) (McDaniel, Jarvis, Donnenberg, & Kaper, 1995; McDaniel & Kaper, 1997), which also contains all the genes necessary to assemble a functional T3SS (Jarvis et al., 1995; Pallen, Beatson, & Bailey, 2005). In addition to the seven effectors encoded in the LEE, there are many others encoded by genes scattered through the genome (Nles: Non‐LEE encoded effectors) that are also translocated by the T3SS (Dean & Kenny, 2009; Deng et al., 2012; Iguchi et al., 2009).

The injectisome of EPEC consists of an outer (EscC) and a pair of inner (EscJ and EscD) membrane rings that are interconnected through a periplasmic inner rod (EscI), forming a core structure, the so‐called basal body, that spans both bacterial membranes (Ogino et al., 2006; Sal‐Man, Deng, & Finlay, 2012; Spreter et al., 2009; Yip et al., 2005). The export apparatus resides within the inner membrane ring and is formed by five highly conserved proteins, named EscR, EscS, EscT, EscU, and EscV (Moraes, Spreter, & Strynadka, 2008). The cytoplasmic side of the basal body is associated to a ring‐shaped oligomeric structure (EscQ and EscK), which functions as a substrate recognition platform, and provides a docking site for the ATPase complex (EscN, EscO, and EscL) (Andrade, Pardo, Espinosa, Perez‐Hernandez, & Gonzalez‐Pedrajo, 2007; Biemans‐Oldehinkel, Sal‐Man, Deng, Foster, & Finlay, 2011; Romo‐Castillo et al., 2014; Soto et al., 2017; Zarivach et al., 2008). Moreover, the extracellular part of the injectisome comprises a 23 nm in length needle‐like structure (EscF), which is extended by a long filament (EspA) (Knutton et al., 1998; Monjaras Feria et al., 2012; Ogino et al., 2006; Sekiya et al., 2001; Wilson, Shaw, Daniell, Knutton, & Frankel, 2001). Upon host cell contact, the filament serves as a scaffold for the assembly of the translocation pore (EspB and EspD) (Chatterjee, Caballero‐Franco, Bakker, Totten, & Jardim, 2015; Luo & Donnenberg, 2011).

Notably, although there is a temporal regulation of LEE gene expression in which the *LEE1* operon is expressed first, the genes encoding all secreted proteins are expressed simultaneously (Yerushalmi, Litvak, Gur‐Arie, & Rosenshine, 2014). Therefore, T3S regulators are involved in setting up a hierarchy of secretion to ensure that the structural proteins that make up the T3SS are secreted prior to effectors (Gaytan et al., 2016; Portaliou, Tsolis, Loos, Zorzini, & Economou, 2016). Depending on the timing of secretion, the T3SS‐dependent substrates are classified as early (EscI and EscF), middle or translocators (EspA, EspB, and EspD), and late substrates or effectors (Deane, Abrusci, Johnson, & Lea, 2010). Coordinated secretion of middle and late substrates has been suggested to be controlled by the LEE‐encoded proteins SepL and SepD (Deng et al., 2004, 2005; O'Connell et al., 2004; Wang, Roe, McAteer, Shipston, & Gally, 2008). Deletion of *sepL* or *sepD* completely abolishes translocator secretion and significantly increases effector secretion (Deng et al., 2004, 2005; Wang et al., 2008). SepL belongs to a family of proteins whose members include MxiC from *Shigella* (Botteaux, Sory, Biskri, Parsot, & Allaoui, 2009), InvE and SsaL from *Salmonella* pathogenicity islands 1 and 2*,* respectively (Coombes, Brown, Valdez, Brumell, & Finlay, 2004; Kubori & Galan, 2002), CopN from *Chlamydia* (Silva‐Herzog et al., 2011)*,* YopN/TyeA from *Yersinia* (Forsberg, Viitanen, Skurnik, & Wolf‐Watz, 1991; Iriarte et al., 1998), and PopN/Pcr1 from *Pseudomonas* (Yang et al., 2007). These proteins, known as gatekeepers, prevent premature effector secretion before host cell contact is established. In most systems, the regulatory function of gatekeepers relies on their ability to disengage from the T3SS base once there is an activation signal upon host cell contact, and subsequently either be secreted, as is the case of YopN, PopN, CopN, and MxiC (Archuleta & Spiller, 2014; Botteaux et al., 2009; Cheng, Kay, & Schneewind, 2001; Cherradi et al., 2013; Lee, Zmina, Stopford, Toska, & Rietsch, 2014), or degraded, as is the case of SsaL (Yu, McGourty, Liu, Unsworth, & Holden, 2010). However, although SepL has been shown to contain a T3S‐signal at its N‐terminal domain, the full‐length protein is not secreted (Deng et al., 2004, 2005; Younis et al., 2010).

SepL forms a trimeric complex with SepD and CesL (Younis et al., 2010). SepD has been proposed to be a homolog of SpiC from *Salmonella* SPI‐2, and CesL a homolog of SycN from *Yersinia*, and both SpiC and SycN are known to be involved in regulation of secretion hierarchy (Cheng et al., 2001; Day & Plano, 1998; Schubot et al., 2005; Younis et al., 2010; Yu, Liu, & Holden, 2004; Yu et al., 2010). Moreover, CesL was suggested to function as a SepL chaperone (Younis et al., 2010), and deletion of the *cesL* (*orf12*) gene in the A/E pathogen *Citrobacter rodentium* abolishes secretion of both translocators and effectors (Deng et al., 2004). Thus, the SepL/SepD/CesL complex resembles the YopN‐TyeA/YscB/SycN complex from *Yersinia* and the SsaL/SpiC/SsaM complex from *Salmonella* SPI‐2 (Younis et al., 2010). These complexes trigger effector secretion by different mechanisms. In the case of *Yersinia*, substrate secretion switching occurs upon YopN secretion, whereas in *Salmonella* it requires the switch protein complex dissociation and the subsequent degradation of its components. In both cases, the switching event occurs in response to environmental cues detected upon host membrane contact, for example, calcium depletion and a pH change, respectively (Cheng et al., 2001; Day & Plano, 1998; Yu et al., 2010). In EPEC, calcium depletion from the bacterial growth media has an effect on substrate secretion similar to that observed in a *sepL* or *sepD* null mutants (Deng et al., 2005; Ide, Michgehl, Knappstein, Heusipp, & Schmidt, 2003). Yet, the molecular mechanism by which the switch protein complex participates in the regulation of substrate secretion in the absence of calcium is still poorly understood. In this work, we investigated the effect of calcium depletion in the gatekeeper‐dependent triggering of effector secretion and uncovered the existence of novel protein interactions.

## 2 MATERIALS AND METHODS

### 2.1 Bacterial strains and growth conditions

All bacterial strains and plasmids used in this study are listed in Table 1. For overnight (O/N) cultures, bacteria were aerobically grown in Lysogeny Broth (LB) at 37°C with constant shaking at 250 rpm. Bacterial growth conditions required for induction of T3S and overproduction of recombinant proteins are indicated below. When necessary, bacterial cultures were supplemented with the appropriate antibiotics at the following concentrations: streptomycin (Sm, 25 μg/ml), kanamycin (Km, 50 μg/ml), ampicillin (Ap, 100 μg/ml), chloramphenicol (Cm, 25 μg/ml), or tetracycline (Tc, 25 μg/ml).

**Table 1:** Strains and plasmids used in this study

Strain/plasmid	Description	Reference
***Escherichia coli***		
EPEC E2348/69	WT EPEC O127:H6 strain; Sm^r^	Levine et al. (1978)
∆*escN* mutant	E2348/69 carrying an in‐frame deletion of *escN*; Sm^r^	Gauthier et al. (2003)
∆*espA* mutant	E2348/69 carrying an in‐frame deletion of *espA*; Sm^r^ Km^r^	Gift of the Navarro F. Lab
∆*espB* mutant	E2348/69 carrying an in‐frame deletion of *espB*; Sm^r^ Km^r^	Gift of the Xicohtencatl J. Lab
∆*espD* mutant	E2348/69 carrying an in‐frame deletion of *espD*; Sm^r^ Km^r^	Gift of the Xicohtencatl J. Lab
∆*sepL* mutant	E2348/69 carrying an in‐frame deletion of *sepL*; Sm^r^	Gift of the Puente JL Lab
∆*escU* mutant	E2348/69 carrying an in‐frame deletion of *escU*; Sm^r^ Km^r^	Soto et al. (2017)
JPEP39 (∆*grlR* mutant)	E2348/69 carrying an in‐frame deletion of *grlR*; Sm^r^	Garcia‐Angulo et al. (2012)
EPEC *sepL*‐3FLAG::km	E2348/69 expressing 3‐FLAG‐tagged *sepL*; Sm^r,^ Km^r^	This study
EPEC *cesL*‐2HA::km	E2348/69 expressing 2‐HA‐tagged *cesL*; Sm^r,^ Km^r^	This study
EPEC *sepL*‐3FLAG *cesL*‐2HA::km	E2348/69 expressing 3‐FLAG‐tagged *sepL* and 2‐HA‐tagged *cesL*; Sm^r,^ Km^r^	This study
BL21(DE3)/pLysS	Strain used for expression of pET19b constructs; Cm^r^	Novagen
XL1‐Blue	Strain used for cloning; Tc^r^	Stratagene
***Salmonella***		
JR501	Strain used to convert plasmids to *Salmonella* compatibility	Ryu & Hartin (1990)
SJW1368	Strain used for expression of pTrc99A_FF4 constructs; flagellar master operon mutant, ∆(cheW‐flhD)	Ohnishi et al. (1994)
**Plasmids**		
pTrc99A_FF4	Modified pTrc99A expression vector under the control of the trc promoter; Ap^r^	Ohnishi, Fan, Schoenhals, Kihara, & Macnab (1997)
pET19b	Expression vector under the control of the T7 promoter; Ap^r^	Novagen
pACTrc	Expression vector under the control of the trc promoter; Cm^r^	Gift of the Fraser GM Lab
pKD46	Red recombinase system plasmid under the control of the *araB* promoter; Ap^r^	Datsenko & Wanner (2000)
pSUB11	Template plasmid for amplification of 3‐FLAG‐kanamycin‐resistance cassette; Ap^r^, Km^r^	Uzzau et al., (2001)
pSU315	Template plasmid for amplification of HA‐kanamycin‐resistance cassette; Ap^r^, Km^r^	Uzzau et al. (2001)
pFLP2	Flp recombinase expression plasmid; Ap^r^	Hoang et al. (1998)
pMTpL	*sepL* cloned into pTrc99A_FF4	This study
pMTpL_∆C75_	*sepL* lacking codons 277 to 351 cloned into pTrc99A_FF4	This study
pMTpL_∆C11_	*sepL* lacking codons 341 to 351 cloned into pTrc99A_FF4	This study
pKEeVc	*escV* codons 335 to 675 cloned into pET19b	This study
pKTeD_N_	*escD* codons 1 to 120 cloned into pTrc99A_FF4	This study
pKEeD_N_	*escD* codons 1 to 120 cloned into pET19b	This study
pJEeI	*escI* cloned into pET19b	Monjaras Feria et al. (2012)
pETeI	*escI* cloned into pTrc99A_FF4	This study
pSLo4	*escK* cloned into pMAL‐c2x	Soto et al. (2017)
pJHeH	*espH* with its native RBS cloned into pTOPO‐2HA	Monjaras Feria et al. (2012)
pJHnC	*nleC* with its native RBS cloned into pTOPO‐2HA	Monjaras Feria et al. (2012)
pJHeI	*escI* with its native RBS cloned into pTOPO‐2HA	Monjaras Feria et al. (2012)
pJHnH2	*nleH2* with its native RBS cloned into pTOPO‐2HA	Monjaras Feria et al. (2012)
pATpD	*sepD* cloned into pTrc99A_FF4	This study
pMEcL	*cesL* cloned into pET19b	This study
pMTBISpDcL	*sepD* and *his‐cesL* cloned into pTrc99A_FF4	This study
pMATpL	*sepL* cloned into pACTrc	This study
pMATpL_∆C11_	*sepL* lacking codons 341 to 351 cloned into pACTrc	This study

### 2.2 Plasmid construction and oligonucleotides

The sequences of the oligonucleotides used in this study are listed in Table 2. The *sepL, sepD,* and *cesL* genes were amplified from chromosomal DNA of the wild‐type EPEC strain E2348/69 (WT), using the following primer pairs: sepL_Fw/sepL_Rv, sepL_Fw/sepL∆C75_Rv, sepL_Fw/sepL∆C11_Rv sepD_Fw/sepD_Rv, and cesL_Fw/cesL_Rv. The resulting PCR products containing the NdeI and BamHI restriction sites were cloned into pTrc99A_FF4 to generate plasmids pMTpL, pMTpL_∆C75_, pMTpL_∆C11_, and pATpD, or into pET19b to generate plasmid pMEcL. To construct plasmids pMATpL and pMATpL_∆C11_, *sepL* or *sepL* lacking the last 11 codons were subcloned from plasmids pMTpL and pMTpL_∆C11_, respectively, into the NdeI/BamHI sites of the pACTrc vector. The pKEeVc plasmid, encoding the cytoplasmic region of EscV, was constructed using primers escV_CtermFw and escV_CtermRv, to amplify a PCR fragment that was cloned into the NdeI/BamHI sites of the pET19b vector. To generate the bicistronic plasmid pMTBISpDcL, the pMEcL plasmid was double digested with XbaI and PstI, and the resulting fragment was cloned into the XbaI/PstI sites of the pATpD plasmid. The pKTeD_N_ plasmid was constructed by amplifying the cytoplasmic region of EscD (codons 1 to 120) using primers escD_N__Fw and escD_N__Rv. The obtained PCR fragment was cloned into the NdeI/BamHI sites of the pTrc99A_FF4 vector. To generate plasmid pKEeD_N_, the pKTeD_N_ construct was double digested with NdeI and BamHI and the resulting fragment was cloned into pET19b. The pJEeI plasmid (Monjaras Feria et al., 2012) was double digested with NdeI and BamHI, and the resulting fragment was cloned into the NdeI/BamHI sites of the pTrc99A_FF4 vector to generate plasmid pETeI. DNA fragments cloned from PCR‐amplified material were sequenced to verify that no undesired base changes had been introduced.

**Table 2:** Oligonucleotides used in this study

Oligonucleotide	Sequence 5′ – 3′
sepL_Fw	AGTTTCATATGGCTAATGGTATTG
sepL_Rv	CTATAAAAAAAAGGATCCTCACAT
sepL∆C75_Rv	TAGCATGGATCCTCAAATGACATC
sepL∆C11_Rv	AATCTATGGATCCTCAAATCATTA
sepD_Fw	TAATACATATGAACAATAATAATG
sepD_Rv	AAAAACTTATTGGATCCATTACAC
cesL_Fw	AGAGCCTGCATATGAATCTTTTAG
cesL_Rv	ATTTAAGAGGATCCTCATGATGTC
escV_CtermFw	AATAATAAGGATCATATGGGAGCTGATTTG
escV_CtermRv	GTGGGTATGGATCCAATACAGAATC
escD_Fw	GGATGAATAAAATTTACATATGTTATCCTCATATAA
escD___Nterm_Rv	CTCGCCAGGATCCGGCGTTATTTGC
sepL‐3FLAG_Fw	ATACATTATTAATGATTGGTAAAGTGATAGATTATAAGGAGGATGTTATGGACTACAAAGACCATGACGG
sepL‐FLAG_Rv	CCTCTTCATAATCTTTCTTAGCATGACAAAAACTATAAAAAAAAACAATAATGAATATCCTCCTTAGTTC
cesL‐2HA_Fw	CTTTTCAACAGCATGTGCAGATTATTGAGCGCGTTCGCAGGATGACATCATATCCGTATGATGTGCCGGACTATGCGTATCCGTATGATGTTCCTGAT
cesL‐2HA_Rv	AAGATCGTGATATGACTCTGCTTTTTTAAATATATTTAAGAGTTTATTCATATGAATATCCTCCTTAGTTC

### 2.3 Epitope tagging of chromosomal genes

A modification of the λ‐Red recombinase system was used for chromosomal epitope tagging as described in (Uzzau, Figueroa‐Bossi, Rubino, & Bossi, 2001). The EPEC strains expressing a 3xFLAG‐tagged version of *sepL* (EPEC *sepL*‐3FLAG::km) or a 2xHA version of *cesL* (EPEC *cesL*‐2HA::km), were constructed by amplifying the kanamycin cassette from either plasmid pSUB11, using the primer pair sepL‐3FLAG_Fw and sepL‐3FLAG_Rv, or from plasmid pSU315, using the primer pair cesL‐2HA_Fw and cesL‐2HA_Rv. The resulting PCR products were electroporated into EPEC WT strain carrying plasmid pKD46, which was grown at 30°C in LB medium containing 100 mmol/L of L‐arabinose to induce Red recombinase expression. Transformant cells were grown at 37°C to eliminate the pKD46 plasmid. Recombinant EPEC *sepL*‐3FLAG::km and EPEC *cesL*‐2HA::km strains were selected on LB plates supplemented with 300 μg/mL Km and tested for Ap sensitivity. To generate the double‐tagged EPEC *sepL*‐3FLAG *cesL*‐2HA::km strain, the kanamycin cassette of the EPEC *sepL*‐3FLAG::km strain was excised using the helper plasmid pFLP2 (Hoang, Karkhoff‐Schweizer, Kutchma, & Schweizer, 1998), and the homologous recombination of *cesL*‐2HA::km was performed as described above.

### 2.4 Type III protein secretion assay

To induce EPEC T3S, preequilibrated regular Dulbecco's modified Eagle's medium (DMEM, Gibco, 12100‐046) was inoculated with 1/100 of an overnight (O/N) culture of LB and grown under static conditions at 37°C in a 5% CO_2_ atmosphere. At an optical density at 600 nm (OD_600_) of 0.8 to 1, the culture was centrifuged (19,800*g* for 10 min) to separate secreted proteins from the bacterial cells. The resulting pellet was resuspended in SDS‐PAGE sample buffer and normalized according to the OD_600_. The supernatant, containing secreted proteins, was precipitated overnight at 4°C with 10% trichloroacetic acid. Precipitated proteins were collected by centrifugation at 19,800*g* for 30 min, and protein pellets were resuspended in SDS‐PAGE sample buffer, normalized according to the OD_600_ and neutralized by adding a saturated Tris solution. Protein secretion profiles were analyzed by 15% SDS‐PAGE and immunoblotting. Calcium‐free DMEM (Gibco, 21068‐028) was used to evaluate the effect of calcium on the EPEC secretion profile. The calcium‐free DMEM solution was supplemented with L‐Glutamine and HEPES to a final concentration of 4 mmol/L and 100 mmol/L, respectively.

### 2.5 Immunoblotting

Samples subjected to SDS‐PAGE were transferred onto nitrocellulose or polyvinylidene fluoride (PVDF) membranes and blocked O/N at 4°C with Tris‐buffered saline (TBS; 20 mmol/L Tris‐HCl, pH 7.5, 150 mmol/L NaCl) containing 0.1% (v/v) Tween 20 and 5% (w/v) nonfat dry milk. After washing with TBS‐Tween, membranes were probed against rabbit‐raised polyclonal anti‐Tir, anti‐Map, anti‐EspA, anti‐EscJ, anti‐EspD, anti‐EscI, anti‐SepD, or anti‐SepL antibodies, and monoclonal anti‐DnaK (MBL International), anti‐FLAG (SIGMA) or HRP‐conjugated anti‐HA (Roche) antibodies. Secondary antibodies used in this study (horseradish peroxidase (HRP)‐conjugated goat antirabbit and goat antimouse), were obtained from Santa Cruz Biotechnology. Protein detection was performed with the Immobilon Western Chemiluminescent HRP Substrate kit (Millipore). Animals were handled and cared for in accordance with the NIH Guide for the Care and Use of Laboratory Animals (A5281‐01) and with the approval of the local Animal Use and Care Committee (CICUAL) of the Instituto de Fisiología Celular, UNAM for protocol BGP70‐15.

### 2.6 Protein overproduction and pull‐down assays

*Salmonella* SJW1368 strain, which lacks the flagellar master operon and has been extensively used for expression of pTrc99A‐based plasmids (Gonzalez‐Pedrajo, Minamino, Kihara, & Namba, 2006; Ohnishi, Ohto, Aizawa, Macnab, & Iino, 1994; Okabe, Minamino, Imada, Namba, & Kihara, 2009), was transformed with plasmids pMTpL, pMTpL_∆C75_, or pMTpL_∆C11_ and *E. coli* BL21 (DE3)/pLysS (BDP) strain with plasmid pKEeVc, for protein overproduction. O/N cultures of the transformed strains were used to inoculate fresh LB containing appropriate antibiotics, and cultures were grown at 37°C with shaking. At an OD_600_ of 0.8, protein production was induced by the addition of isopropyl β‐D‐1‐thiogalactopyranoside (IPTG) to a final concentration of 0.1 mmol/L, and bacterial growth was continued for 4 hr at 30°C. The bacterial cells were harvested by centrifugation at 15,500*g* for 10 min, and the pellets were resuspended in binding buffer (20 mmol/L Tris‐HCl pH 8, 0.5 mol/L NaCl) containing 20 mmol/L imidazole and 1 mmol/L phenylmethylsulfonyl fluoride (PMSF) and lysed by sonication. Cell lysates were centrifuged (15,500*g* for 30 min) and the supernatants (cleared lysates) containing soluble proteins were recovered. The cleared lysate of the His‐EscVc recombinant protein was mixed with the cleared lysate of SepL, SepL_∆C75_, or SepL_∆C11_ and incubated for 2 hr at 4°C. Mixed lysates were loaded onto a polypropylene column (QIAGEN) containing 100 μl of preequilibrated Ni‐nitrilotriacetic acid (Ni‐NTA) agarose beads. The resin was washed with 10 column volumes of binding buffer containing increasing concentrations of imidazole and bound proteins were eluted with 500 mmol/L imidazole. To purify the SepL/SepD/CesL protein complex, *Salmonella* SJW1368 cells were cotransformed with the bicistronic plasmid pMTBISpDcL and the plasmid pMATpL or pMATpL_∆C11_. Protein production was induced by the addition of 1 mmol/L IPTG. The cleared lysate was prepared as described above, incubated with 100 μL of Ni‐NTA for 2 hr at 4°C and then loaded onto a polypropylene column. After extensive washing, proteins were eluted with 500 mmol/L imidazole. To assess the effect of calcium on the EscVc/SepL and SepL/SepD/CesL interactions, 2 mmol/L CaCl_2_ was added to all buffers used in the pull‐down assays.

To evaluate the interaction of EscVc and EscD_N_ with T3‐secreted proteins, a modified version of the method employed by Thomas et al. (2005) to identify cognate substrates of the CesT chaperone was used. Briefly, the cleared lysate of His‐EscVc or His‐EscD_N_ was incubated with Ni‐NTA agarose beads for 2 hr and the resin was packed into a polypropylene column. After washing with 20 mmol/L imidazole‐binding buffer, the mixed supernatant of mutant strains ∆*sepL* and ∆*grlR* grown under T3S‐inducing conditions was loaded onto the column, washed with five column volumes of binding buffer containing increasing concentrations of imidazole, and eluted with 500 mmol/L imidazole. All resulting samples were subjected to SDS‐PAGE and immunoblotting.

### 2.7 Subcellular fractionation

Subcellular fractionation was carried out as previously reported (Gauthier, Puente, & Finlay, 2003) with slight modifications. The EPEC *sepL*‐3FLAG *cesL*‐2HA::km strain was grown under T3S inducing conditions in regular DMEM or DMEM without calcium until an OD_600_ of 1. Cells were harvested by centrifugation (15,500*g* for 10 min), the cell pellet was washed once with phosphate‐buffered saline and then resuspended in 20 mmol/L Tris‐HCl pH 7.5 containing 1 mmol/L PMSF. Cells were disrupted by sonication and unbroken cells were removed by centrifugation at 19,800*g* for 10 min. The cleared lysate was subjected to ultracentrifugation at 90,000*g* for 1 hr. The supernatant (containing the cytoplasmic proteins) was collected into a clean tube, and the membrane‐containing pellet was washed with 20 mmol/L Tris‐HCl pH 7.5. Both fractions were ultracentrifuged once again as previously described, and the cytoplasmic and membrane fractions were collected. Total protein concentration of the samples was quantified using the DC protein assay (Bio‐Rad), and proteins were analyzed by immunoblotting as described above.

### 2.8 Protein stability assay

The EPEC *sepL*‐3FLAG *cesL*‐2HA::km strain was grown in calcium‐free DMEM added with 1.8 mmol/L CaCl_2_ under T3S inducing conditions. When an OD_600_ of 1 was reached, cells were harvested by centrifugation at 4,600*g* for 10 min, washed with and resuspended in either calcium‐free DMEM or calcium‐free DMEM added with 1.8 mmol/L CaCl_2_, containing 50 μg/ml Cm in order to stop protein synthesis. Bacterial growth was continued and samples were taken every 30 min during 3 hr. Samples were normalized according to the OD_600_ and proteins were analyzed by immunoblotting as described above.

### 2.9 Coimmunoprecipitation

50 mL of DMEM without calcium or the same medium added with 1.8 mmol/L CaCl_2_ was inoculated with O/N cultures of the EPEC *sepL*‐3FLAG *cesL*‐2HA::km or EPEC *cesL*‐2HA::km strains. Cell cultures were grown under static conditions at 37°C in a 5% CO_2_ atmosphere. At an OD_600_ of 1, cultures were harvested by centrifugation, cell pellets were washed with HEPES 20 mmol/L, NaCl 250 mmol/L pH 7.4, resuspended in the same buffer and lysed by sonication. Cell lysates were incubated with Triton X‐100 [0.1% (v/v)] for 15 min and then cleared by centrifugation at 19,800*g* for 30 min. Supernatants were carefully collected and the crosslinker dithiobis succinimidyl propionate was added to a final concentration of 1 mmol/L. After 1 hr incubation at room temperature, the crosslinking reaction was stopped by the addition of 20 mmol/L Tris pH 7.5. Cross‐linked samples were mixed with 40 μl of preequilibrated ANTI‐FLAG M2 Affinity Gel beads (SIGMA) and incubated O/N at 4°C with shaking. SepL‐3FLAG coupled beads were centrifuged at 13,500*g* for 2 min and washed three times with HEPES 20 mmol/L, NaCl 250 mmol/L pH 7.4. Finally, the beads were resuspended in 20 μl of SDS‐PAGE sample buffer, containing 2 μl of β‐mercaptoethanol and boiled for 5 min. Protein samples were resolved by SDS‐PAGE and analyzed by immunoblotting as described above.

## 3 RESULTS

### 3.1 Calcium removal from the culture media solely affects effector secretion

**Figure 1:**
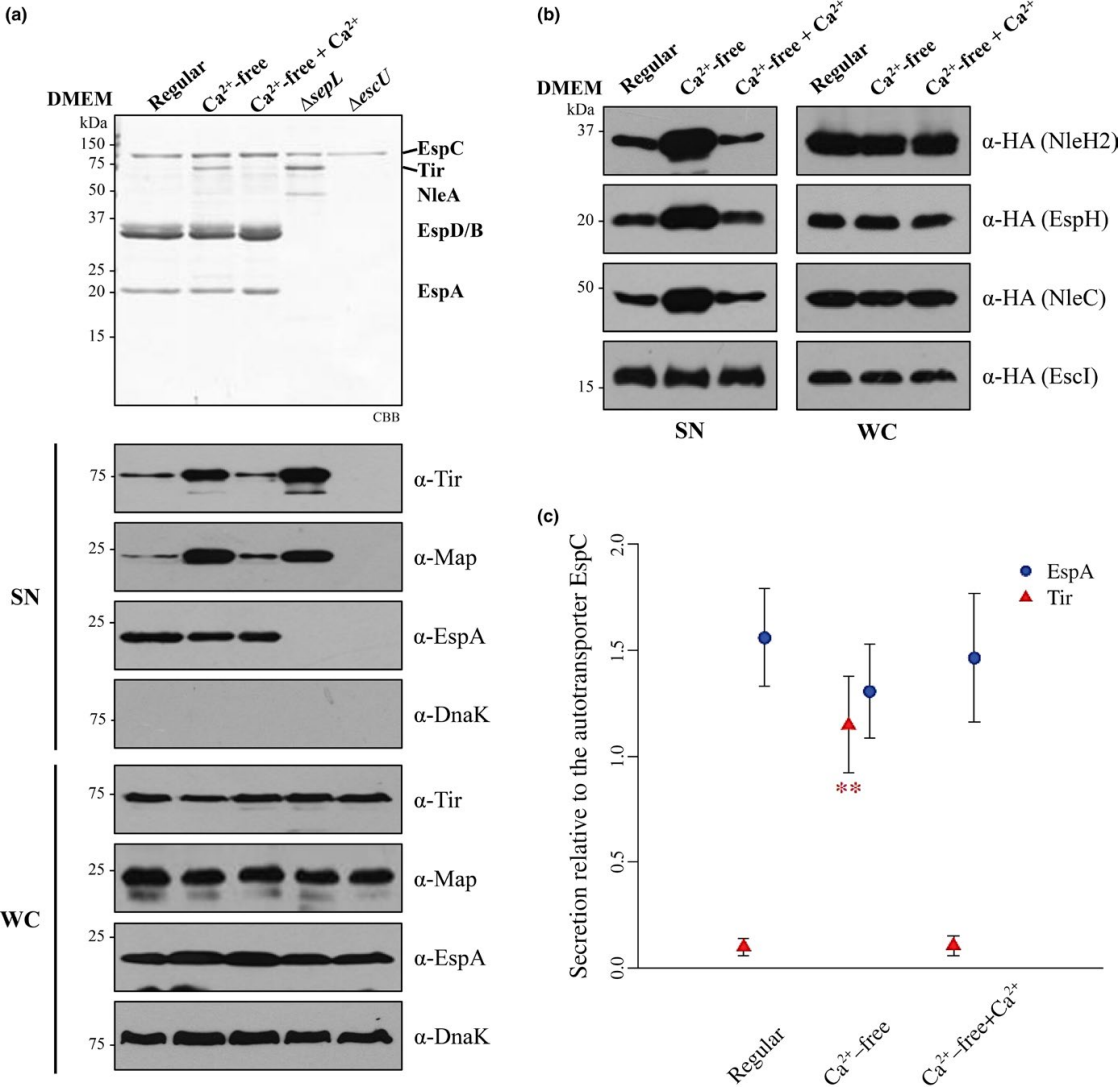
Calcium depletion from the bacterial growth media enhances effector secretion. (a) T3 secretion profiles of the EPEC wild‐type strain (WT) grown in regular DMEM containing 1.8 mmol/L of CaCl_2_ (Regular), calcium‐free DMEM (Ca^2+^‐free) or calcium‐free DMEM supplemented with 1.8 mmol/L of CaCl_2_ (Ca^2+^‐free + Ca^2+^), and the ∆*sepL* and ∆*escU* mutant strains, visualized by SDS‐PAGE stained with Coomassie brilliant blue (CBB) (upper panel). The presence of DnaK, Tir, Map, and EspA in the supernatants (SN) and whole‐cell lysates (WC) was examined by immunoblotting, using anti‐DnaK, anti‐Tir, anti‐Map, and anti‐EspA antibodies (lower panels). (b) Protein secretion profiles of EPEC WT strain overproducing HA‐tagged T3 substrates, grown in the presence or absence of calcium as described in (a). Immunodetection of LEE and non‐LEE‐encoded effectors (NleH2, EspH, and NleC) and the inner rod protein EscI, was performed in the supernatants (SN) and whole‐cell lysates (WC) using specific antibodies against the HA tag. The results shown are representative of three independent experiments. (c) Relative abundance of EspA and Tir proteins in the supernatant of EPEC wild‐type strain grown in the presence or absence of calcium as described in (a). CBB‐stained protein bands were quantified from six independent secretion assays by gel densitometry using the ImageJ software (Schneider, Rasband, & Eliceiri, 2012). The secretion level of EspA and Tir proteins was normalized relative to the secretion level of the EspC autotransporter band. The average and the standard deviation of normalized data are displayed. Significant statistical differences compared with the regular DMEM condition are denoted by asterisks. A *p* value < .05 was considered statistically significant. *^*^ *p* = .002, Wilcoxon‐test

Previous studies have implicated calcium as an important player in the regulation of T3 substrate secretion in EPEC (Deng et al., 2005; Ide et al., 2003; Kenny, Abe, Stein, & Finlay, 1997; Shaulov, Gershberg, Deng, Finlay, & Sal‐Man, 2017). Therefore, we examined the secretion profile of EPEC wild‐type strain E2348/69 grown under T3S inducing conditions in regular DMEM (containing 1.8 mmol/L CaCl_2_) or calcium‐free DMEM. We confirmed that the secretion of the effector Tir is increased in the absence of calcium (Figure 1a), and extended this finding to other LEE and Nle effectors as judged by the enhanced secretion of Map, EspH‐2HA, NleH2‐2HA, and NleC‐2HA into the supernatant (Figure 1a and b). Addition of 1.8 mmol/L CaCl_2_ to calcium‐free DMEM restored effector secretion to the levels seen with regular DMEM (Figure 1), corroborating that the observed effect is due to the absence of calcium. For comparison, the secretion profiles of the *sepL* null mutant, which entirely deregulates effector secretion, and that of the secretion‐deficient *escU* null mutant, are shown (Figure 1a). In addition, our results demonstrated that protein production was not affected by the different culture media used or in the mutant strains (Figure 1a lower panel and b right panel). Furthermore, in contrast to previous reports (Deng et al., 2005; Ide et al., 2003), we did not observe a significant decrease in translocator secretion (Figure 1a). Therefore, to provide a more quantitative measure of the overall change in the secretion of effectors and translocators, we performed a statistical analysis on the secretion data of several independent experiments by quantifying the Coomassie‐stained protein band intensities of the translocator EspA and the effector Tir. The values were normalized relative to the corresponding band intensity of the autotransporter EspC, which is secreted through the type V secretion system and thus serves as a loading control (Mellies et al., 2001; Stein, Kenny, Stein, & Finlay, 1996). This analysis showed no significant differences in EspA translocator secretion in the different media used in the assay, whereas there was a clear 11‐fold increase in Tir effector secretion in the absence of calcium (Figure 1c). Moreover, we investigated the effect of calcium on the secretion of an early substrate, namely, the inner rod component EscI. As shown in Figure 1b, secretion of EscI‐2HA was not altered by different calcium levels in the growth media. It is worth mentioning that although overproduction of EscI‐2HA caused a decrease in T3 secretion, EPEC was still responsive to calcium depletion as demonstrated by an increased secretion of Tir (Figure S1A). Besides, overproduction of an untagged version of EscI, which did not interfere with T3 secretion, further demonstrated that calcium levels had no effect on the secretion of this early substrate (Figure S1B). Overall, our results suggest that only late substrate secretion is significantly affected by calcium depletion from the growth media. Thus, we next decided to investigate the mechanism that triggers effector secretion under this condition.

### 3.2 Calcium sensing is translocon‐independent

**Figure 2:**
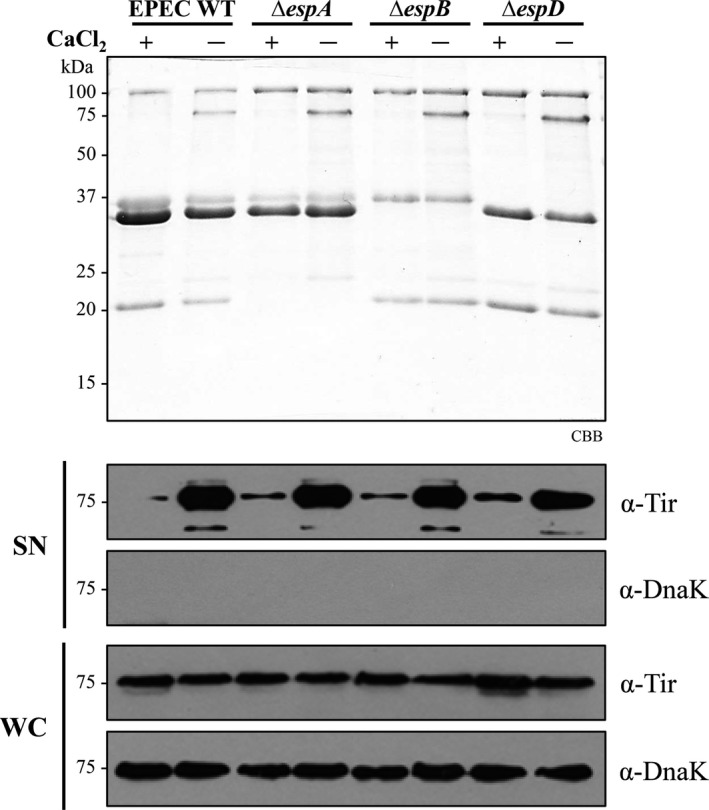
The translocon components EspA, EspB, and EspD are dispensable for calcium sensing. The secretion profiles of the EPEC wild‐type strain (WT) and the ∆*espA*, ∆*espB,* and ∆*espD* null mutants grown under T3S inducing conditions in the presence or absence of calcium were analyzed by Coomassie brilliant blue stained SDS‐PAGE (upper panel). Immunodetection of secreted proteins (SN) and whole‐cell lysates (WC) was performed using specific antibodies against Tir and DnaK (bottom panel)

It has been previously shown that effector secretion is elicited upon host cell contact, and that different environmental cues are involved in the activation of this secretion stage, for example, a pH shift in *Salmonella* and low‐calcium in *Yersinia*,* Pseudomonas,* and A/E pathogens (Beuzon, Banks, Deiwick, Hensel, & Holden, 1999; Deng et al., 2005; Kim et al., 2005; Lee, Mazmanian, & Schneewind, 2001; Mills, Baruch, Charpentier, Kobi, & Rosenshine, 2008; Rosqvist, Magnusson, & Wolf‐Watz, 1994; Yu et al., 2010). These in vitro conditions are proposed to mimic those found during the bacterial infection process. Therefore, we reasoned that the translocon proteins in EPEC, which establish contact with host cells, could be involved in sensing calcium‐level changes. This hypothesis was evaluated by comparing the secretion profile of the EPEC wild‐type strain with that of the isogenic translocator null mutants *espA*,* espB,* and *espD*, when grown in the presence or absence of calcium. Translocator null mutants were still responsive to calcium depletion, as shown by an increased secretion of the effector Tir in calcium‐free medium (Figure 2). Tir production was not affected in the different culture media or in the mutant strains (Figure 2 lower panel). Thus, although translocators are the proteins that make direct contact with host cells, they appear not to be required for in vitro calcium sensing.

### 3.3 The SepL/SepD/CesL complex is insensitive to calcium concentration changes

**Figure 3:**
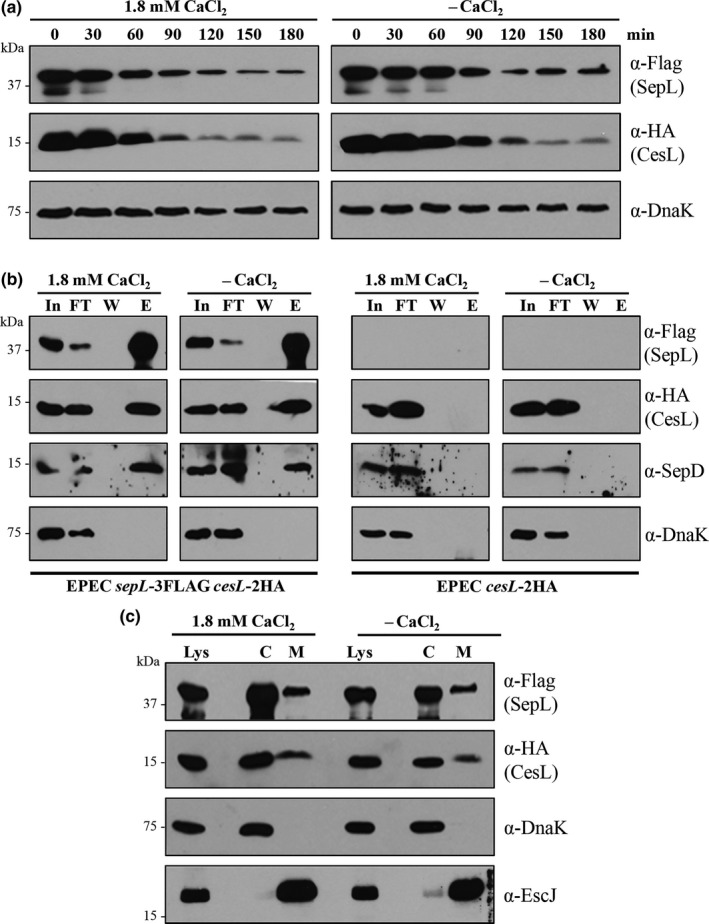
The SepL/SepD/CesL complex does not participate in the calcium‐absence response. (a) Immunoblot analysis of whole‐cell lysates of the EPEC *sepL‐*3FLAG *cesL‐*2HA strain grown under T3S inducing conditions in the presence or absence of calcium. Intracellular protein stability of SepL‐3FLAG and CesL‐2HA was examined every 30 min during 3 hr after the addition of chloramphenicol, by immunodetection using anti‐FLAG and anti‐HA antibodies. Anti‐DnaK was used as a loading control. (b) Immunoprecipitation of SepL‐3FLAG from the EPEC *sepL‐*3FLAG *cesL‐*2HA and EPEC *cesL‐*2HA strains grown in the presence or absence of calcium. Coimmunoprecipitation of SepD, CesL‐2HA, and DnaK with SepL‐3FLAG was corroborated with specific antibodies against SepD, HA‐tag, and DnaK. (c) The EPEC *sepL*‐3FLAG *cesL*‐2HA strain grown in the presence or absence of calcium was fractionated into cytoplasmic (C) and membrane (M) fractions. Immunodetection of SepL‐3FLAG and CesL‐2HA in the cell lysate (Lys) and in the cytoplasmic and membrane fractions was performed using antibodies against the FLAG and HA tags. To confirm proper fractionation, samples were probed with anti‐DnaK and anti‐EscJ antibodies as cytoplasmic and membrane markers, respectively

It has been reported that the SsaL/SpiC/SsaM complex of the *S. enterica* SPI‐2 T3SS prevents premature effector secretion at pH 5.0, a condition found within host cell vacuoles. However, upon T3SS vacuolar membrane contact, the detection of a pH increase to 7.2 in the cytoplasm leads to dissociation of the protein complex and degradation of its components, resulting in effector secretion (Yu et al., 2010). Since the proteins forming the SsaL/SpiC/SsaM complex in *Salmonella* are homologous to the ones forming the SepL/SepD/CesL complex in EPEC, and both protein complexes participate in the substrate specificity switch from translocators to effectors without secretion of the gatekeeper protein (Coombes et al., 2004; Younis et al., 2010; Yu et al., 2010), we argued that the mechanism by which these complexes respond to environmental cues that induce effector secretion could be similar. Therefore, we examined the effect of calcium depletion on the stability, formation, and subcellular localization of the SepL/SepD/CesL complex. To this end, we constructed a *sepL*‐3FLAG *cesL*‐2HA chromosomally tagged strain and showed that its T3 secretion profile is comparable to that of the wild‐type strain (Figure S2). However, we were unable to obtain a functional SepD chromosomally tagged strain, because the C‐terminal tag affected the protein secretion profile (data not shown). Then, we evaluated the effect of calcium depletion on the protein stability of SepL‐3FLAG and CesL‐2HA. Stability assays were performed as described under Materials and Methods, inhibiting de novo protein synthesis with chloramphenicol in cells grown in the presence or absence of calcium. As shown in Figure 3a, the stability profile of SepL and CesL was similar under both conditions tested, indicating that neither of these proteins is degraded in response to a drop in calcium concentration. Subsequently, we immunoprecipitated SepL‐3FLAG from the EPEC *sepL*‐3FLAG *cesL*‐2HA strain grown in the presence or absence of calcium. The results showed that CesL‐2HA and SepD were coprecipitated at similar levels in both conditions (Figure 3b left panel), indicating that calcium removal from the medium does not result in SepL/SepD/CesL complex dissociation. Specificity controls demonstrated that neither the CesL‐2HA nor SepD proteins bound nonspecifically to the anti‐FLAG beads (Figure 3b right panel), and that a non‐T3SS‐related protein, the chaperone DnaK, was not coprecipitated with SepL‐3FLAG and did not bind the anti‐FLAG beads (Figure 3b bottom panels). Furthermore, to test the possibility that the localization of the SepL/SepD/CesL complex, and hence, its association with the T3S base components might be altered, we carried out a subcellular fractionation of the EPEC *sepL*‐3FLAG *cesL*‐2HA strain grown in the presence or absence of calcium. The results showed that both SepL and CesL were similarly distributed in the cytoplasm and cosedimented with the membrane fraction under the two different growth conditions (Figure 3c), indicating that calcium depletion does not affect the localization of the SepL/SepD/CesL complex.

Moreover, it has been suggested that environmental signals can diffuse through the needle channel to a sensor protein at the base of the secretion apparatus (Notti & Stebbins, 2016; Portaliou et al., 2016; Shaulov et al., 2017; Yu et al., 2010). Therefore, we decided to investigate whether calcium directly affected the formation of the SepL/SepD/CesL trimeric complex. To this end, in vitro pull‐down assays were performed in the presence or absence of calcium as described in 2. The results showed no differences in the copurification assays with or without calcium (Figure S3). This is consistent with a previous report showing that calcium did not affect the SepL‐SepD interaction (Deng et al., 2005). Overall, these findings indicate that the mechanism by which effector secretion increases upon calcium removal from the growth media does not involve changes in the stability, formation, or localization of the SepL/SepD/CesL secretion regulatory complex.

### 3.4 SepL interacts with EscV, a T3SS component engaged in substrate recognition

The gatekeeper protein SepL and its homologs in different bacteria have been shown to interact with diverse T3SS components (Archuleta & Spiller, 2014; Botteaux et al., 2009; Cherradi et al., 2013; Kubori & Galan, 2002; Lee et al., 2014; Roehrich et al., 2017; Shen & Blocker, 2016; Wang et al., 2008). On the basis of our previous results, we reasoned that T3SS proteins involved in substrate recognition or targeting might be prone to calcium‐mediated control.

**Figure 4:**
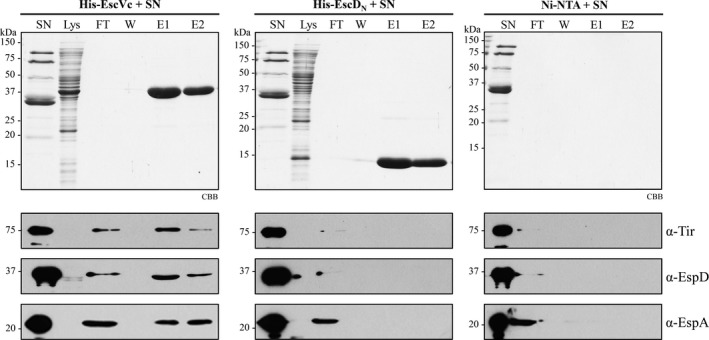
EscVc directly interacts with secreted proteins. Pull‐down assay of His‐EscVc (left panels) and His‐EscD_N_ (middle panels) with secreted proteins, performed by nickel affinity chromatography. The cleared lysate containing His‐EscVc or His‐EscD_N_ (Lys), was incubated with Ni‐NTA beads and loaded into a column. The proteins secreted into the supernatant by the ∆*sepL* and ∆*grlR* null mutant strains (SN) were passed through the His‐EscVc or His‐EscD_N_‐coupled resin in the column and the flow through (FT) was collected. After extensive washing (W), proteins were eluted (E1 and E2). Samples were visualized by SDS‐PAGE stained with Coomassie brilliant blue (CBB) (upper panels). Detection of copurified proteins was performed by immunoblotting with specific antibodies against Tir, EspD, and EspA (lower panels). As an additional control, the nonspecific binding of Tir, EspD, and EspA to the Ni‐NTA beads was analyzed by SDS‐PAGE stained with CBB and immunoblotting (right panels)

Homologous proteins of the EPEC export apparatus component EscV have been implicated in the recruitment of T3 secretion substrates and chaperones, in both the flagellar and virulence T3SSs. In *Xanthomonas spp*., the cytoplasmic domain of HrcV was shown to bind early and late substrates, as well as chaperones (Alegria et al., 2004; Buttner, Lorenz, Weber, & Bonas, 2006; Hartmann & Buttner, 2013), whereas the cytoplasmic domain of the flagellar export gate protein FlhA, binds different chaperone/substrate complexes (Bange et al., 2010; Kinoshita, Hara, Imada, Namba, & Minamino, 2013). To investigate whether EscV might play a role in substrate recognition, we tested the ability of the C‐terminal cytoplasmic domain of EscV (EscVc) to interact with secreted proteins. To this aim, His‐EscVc was coupled to Ni‐NTA agarose beads and incubated with the combined supernatants of the secretion assays of a ∆*sepL* mutant, which hypersecretes effectors, and a ∆*grlR* mutant, which produces and secretes more effectors and translocators (Deng et al., 2004, 2005; Wang et al., 2008). After extensive washing, proteins were eluted and analyzed by SDS‐PAGE and immunoblotting. The results showed that His‐EscVc interacts with both middle (EspA and EspD) and late (Tir) substrates (Figure 4 left panel). As a control, substrate binding to EscD, which is also an inner membrane protein with a cytoplasmic domain (EscD_N_), was evaluated under the same conditions and no interaction was observed (Figure 4 middle panel). In addition, we discarded the possibility of substrate nonspecific binding to the Ni‐NTA resin (Figure 4 right panel). These results suggest that the major export gate component of the EPEC injectisome, EscV, provides a docking site for substrates prior to their T3‐dependent secretion.

**Figure 5:**
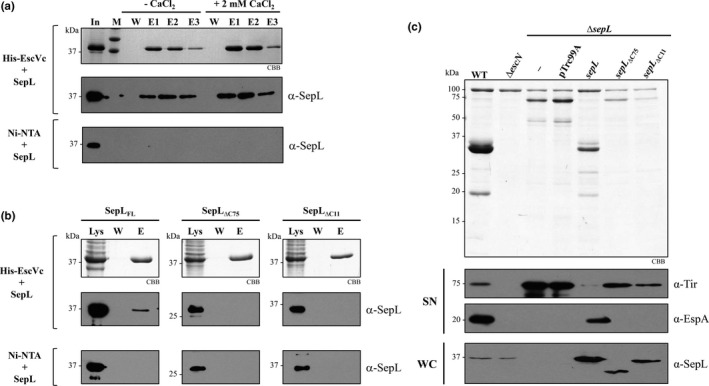
SepL interacts with the export gate component EscV. (a) Pull‐down assays of His‐EscVc and SepL (His‐EscVc + SepL) performed by nickel affinity chromatography in the presence or absence of 2 mmol/L CaCl_2_. Cleared lysates containing His‐EscVc or untagged SepL were mixed and incubated for 2 hr (In) in the absence or presence of calcium. Samples were loaded into columns packed with Ni‐NTA resin, washed (W) and eluted (E1, E2, and E3). His‐EscVc was visualized by SDS‐PAGE stained with Coomassie brilliant blue (CBB). The molecular mass markers (M) of 37 and 50 kDa are shown. Detection of copurified SepL was performed by immunoblotting with polyclonal antibodies against SepL (middle panel). As a negative control, SepL was loaded into a Ni‐NTA column (Ni‐NTA + SepL) and treated under the same conditions described above (lower panel). (b) Pull‐down assays of His‐EscVc and C‐terminal truncated versions of SepL (SepL_∆C75_ and SepL_∆C11_) performed by nickel affinity chromatography. Cleared lysates containing His‐EscVc and full‐length SepL (SepL_FL_), SepL_∆C75_, or SepL_∆C11_ were mixed and incubated for 2 hr (Lys). Samples were loaded into columns packed with Ni‐NTA resin, washed (W), and eluted (E). His‐EscV was visualized by SDS‐PAGE stained with CBB. Copurified proteins were detected by immunoblotting using polyclonal anti‐SepL antibodies (middle panels). As negative controls, the nonspecific binding of SepL_FL_, SepL_∆C75_, and SepL_∆C11_ to the Ni‐NTA resin was analyzed by immunoblotting (lower panels). (c) Protein secretion profiles of EPEC wild‐type (WT), ∆*escN* and ∆*sepL* mutant strains, and the ∆*sepL* strain carrying the empty vector pTrc99A_FF4 (pTrc99A), or the pTrc99A_FF4‐based plasmids expressing *sepL* or the C‐terminal truncated versions *sepL*_∆C75_ and *sepL*_∆C11_. Secreted proteins were visualized by SDS‐PAGE stained with CBB (upper panel) and detected by immunoblotting in the supernatants (SN) with specific antibodies against Tir and EspA. The production of SepL and its truncated versions in whole‐cell lysates (WC) was examined by immunoblotting with polyclonal antibodies against SepL

Next, we investigated a possible link between the gatekeeper SepL and the EscV protein, by evaluating a physical interaction between these proteins and its calcium‐dependence. For this purpose, we performed a nickel affinity copurification assay by mixing the cleared lysates of cells producing His‐EscVc and untagged SepL, in the presence or absence of calcium. Affinity resin‐bound proteins were eluted and analyzed by SDS‐PAGE and immunoblotting. The result showed that SepL directly interacts with the cytoplasmic domain of EscV and that this interaction is not dependent on the presence of calcium (Figure 5a). SepL binding to the Ni‐NTA resin was not detected (Figure 5a lower panel). As an additional control, we showed that under the conditions tested, His‐EscVc does not bind to MBP‐EscK (data not shown), as previously observed in a yeast two hybrid screen (Soto et al., 2017), corroborating the specificity of the EscV‐SepL interaction.

To further dissect the SepL interacting region with EscVc, we generated SepL truncated protein versions. In *P. aeruginosa,* the EscV homolog PcrD interacts with the Pcr1 protein, which is homologous to the C‐terminal domain of SepL (Lee et al., 2014). Therefore, we generated two C‐terminal truncated versions of SepL (SepL_∆C75_ and SepL_∆C11_) and assessed their ability to interact with His‐EscVc by in vitro pull‐down assays. We found that in contrast to full‐length SepL, neither SepL_∆C75 _nor SepL_∆C11_ interacted with His‐EscVc (Figure 5b). In addition, we demonstrated that the structural integrity of the SepL_∆C11_ truncated protein is not affected, since it is able to form the SepL_∆C11_/SepD/CesL ternary complex (Figure S4). These results indicate that the last 11 amino acids of SepL are crucial for its interaction with EscVc. Moreover, we further evaluated the ability of the SepL truncated versions to complement the phenotype of the ∆*sepL* null strain. Neither SepL_∆C75 _nor SepL_∆C11_ could restore the secretion of translocators, and only partially reduced the hypersecretion of effectors of the *sepL* mutant (Figure 5c). Therefore, our results highlight the importance of the extreme C‐terminal region of SepL for substrate secretion regulation.

## 4 DISCUSSION

Injectisome assembly demands orderly secretion of T3 substrates. Secretion of translocators prior to effectors ensures that the latter are translocated directly into the host cell cytoplasm. In EPEC, this secretion hierarchy is proposed to be controlled by the gatekeeper SepL (Deng et al., 2004, 2005; Wang et al., 2008), and to respond to environmental cues such as calcium concentration (Deng et al., 2005; Shaulov et al., 2017). However, the exact mechanism underlying this specificity‐switching process is still poorly understood. In this work, we evaluated the link between calcium sensing and SepL hierarchical regulation.

Previous reports showed that calcium depletion from the growth medium in A/E pathogens has a differential effect on T3 substrate secretion, that is, reduced secretion of translocators and increased secretion of effectors (Deng et al., 2005; Ide et al., 2003). In accordance, we showed that the absence of calcium in the EPEC growth medium considerably increased the secretion of several LEE and Nle effectors. However, although the secretion of translocators appears to be slightly reduced in the absence of calcium, our statistical analysis demonstrated that the secretion of middle substrates is not significantly affected under this condition (Figure 1). The dissimilar results on the effect of calcium on translocator secretion could be attributed to either differences in the experimental setups for the secretion assay or to the use of chelators to eliminate calcium (Deng et al., 2005; Ide et al., 2003), given that EGTA and BAPTA bind in addition to calcium other multivalent cations, which in turn could also affect T3‐dependent secretion (Gode‐Potratz, Chodur, & McCarter, 2010; Kenny et al., 1997; Sarty et al., 2012). In agreement with our results, a previous report in *P. aeruginosa* showed that in vitro calcium levels influence effector but not translocator secretion (Cisz, Lee, & Rietsch, 2008). Hence, it is possible that secretion of translocators and effectors can be promoted by different environmental signals as recently suggested (Roehrich et al., 2017). Additionally, we extended our analysis to investigate the effect of calcium absence on the secretion of an early substrate, the inner rod protein EscI, and demonstrated that its secretion is not affected under this condition. This result supports our proposal that the calcium signal exclusively regulates the substrate specificity switch promoting late substrate secretion.

Moreover, it has been proposed that physical contact between the bacteria and host cells along with the assembly of the translocation pore, allows for detection of lowered calcium levels in the eukaryotic cell cytoplasm, triggering effector secretion (Deng et al., 2005; Lee et al., 2001). Accordingly, the translocon components would be expected to be involved in signal sensing as has been previously reported (Armentrout & Rietsch, 2016; Cisz et al., 2008; Roehrich, Guillossou, Blocker, & Martinez‐Argudo, 2013; Urbanowski, Brutinel, & Yahr, 2007). Here we demonstrated that the translocator proteins EspA, EspB, and EspD are not implicated in transducing the signal that triggers effector secretion upon calcium depletion in vitro (Figure 2). This finding is in agreement with previous reports in *S. enterica* and *P. aeruginosa,* where mutants lacking the translocators are still able to trigger effector secretion upon changes in chemical cues (Cisz et al., 2008; Yu et al., 2010). The apparent discrepancy regarding the requirement of translocators could be explained by the nature of the triggering signal. While translocators have been shown to be essential for effector translocation upon host cell contact (Armentrout & Rietsch, 2016; Cisz et al., 2008; Urbanowski et al., 2007)*,* they are not required for induction of effector secretion under calcium depletion in vitro (Cisz et al., 2008; Lee, Stopford, Svenson, & Rietsch, 2010). Thus, under in vitro calcium‐depleted conditions in EPEC, either the needle plays an active role in detecting and conveying the environmental signal as has been demonstrated for *S. flexneri* and *Y. pestis* (Deane et al., 2006; Kenjale et al., 2005; Torruellas, Jackson, Pennock, & Plano, 2005); or as recently suggested, diffusion of chemical signals through the needle channel might alter the local concentration of different ions at the base of the secretion apparatus (Notti & Stebbins, 2016; Portaliou et al., 2016; Shaulov et al., 2017; Yu et al., 2010), resulting in modifications at the T3SS basal machinery that trigger effector secretion. Nonetheless, further studies are required to elucidate the mechanistic basis of physiological signal sensing and transmission.

The deregulation of effector secretion upon calcium depletion somewhat mimics the hypersecretion phenotype seen in the absence of the SepL and SepD proteins (Deng et al., 2005). SepL and SepD form a ternary complex with CesL that is homologous to the *S. enterica* SsaL/SpiC/SsaM complex (Younis et al., 2010). Besides, in contrast to other gatekeeper proteins, SepL and SsaL are not secreted, share more than 40% sequence similarity and SepL partially complements an *ssaL* null mutant (Coombes et al., 2004; Younis et al., 2010). For these reasons, we hypothesized that the SepL/SepD/CesL complex regulates the switch from middle to late substrates in response to environmental calcium in the same manner as the SsaL/SpiC/SsaM complex does in response to pH (Yu et al., 2010). Nevertheless, our results demonstrated that the stability, localization, and association of the SepL/SepD/CesL complex are not affected by calcium changes (Figure 3 and S3), indicating that this gatekeeper complex is not prone to direct or indirect calcium regulation. These data prompted us to investigate whether the interaction with other T3SS components might be involved in the calcium‐signaling pathway.

Gatekeepers are known to interact with several T3SS components (Archuleta & Spiller, 2014; Botteaux et al., 2009; Cherradi et al., 2013; Day & Plano, 1998; Kubori & Galan, 2002; Lee et al., 2014; Roehrich et al., 2017; Shen & Blocker, 2016; Silva‐Herzog et al., 2011; Stone, Johnson, Bulir, Gilchrist, & Mahony, 2008). In EPEC, besides the SepL/SepD/CesL complex, SepL binds to the effector Tir, the inner membrane ring‐forming protein EscD (Wang et al., 2008), and to the switch protein EscP (Shaulov et al., 2017). Among the gatekeepers interacting partners, those involved in substrate recognition are perfect targets for secretion regulation. Substrate docking sites have been found in the cytoplasmic domain of the export apparatus component SctV and its flagellar homolog FlhA (Bange et al., 2010; Hartmann & Buttner, 2013; Kinoshita et al., 2013; Minamino et al., 2012). Noteworthy, in the flagellar T3SS, the distinct binding affinities of FlhA for different substrate/chaperone complexes seem to coordinate the order of substrate secretion (Kinoshita et al., 2013). In this study, we demonstrated that the cytoplasmic domain of the major export gate component EscV (EscVc) is indeed capable of interacting with both middle and late T3 substrates (Figure 4). In addition, we showed that SepL interacts with EscVc, even though the interplay between these components was not regulated directly by calcium (Figure 5a). In this same regard, the SepL homologs in *P. aeruginosa* (PopN/Pcr1) and *S. flexneri* (MxiC) have been found to interact with SctV (PcrD and MxiA, respectively) (Lee et al., 2014; Shen & Blocker, 2016). In agreement with our result, the Pcr1‐PcrD interaction was not sensitive to calcium removal from the growth media (Lee et al., 2014). Interestingly, a single point mutation in PcrD and MxiA that interferes with their binding to Pcr1 and MxiC, respectively, caused a deregulation of effector secretion (Lee et al., 2014; Shen & Blocker, 2016), suggesting that the SctV‐gatekeeper protein interaction is relevant for T3S regulation.

The SepL region involved in the interaction with EscV was mapped to the last 11 amino acids (Figure 5b), and we showed that this protein version, SepL_∆C11_, is unable to rescue the phenotype of a *sepL* null mutant (Figure 5c). This result is similar to previous observations in enterohemorrhagic *E. coli*, where complementation of a *sepL* mutant with a SepL_∆C11_ protein version did not recover the secretion of translocators to wild‐type levels, and only marginally reduced the boosted secretion of effectors (Wang et al., 2008). These results are also in agreement with recent observations in *Shigella* showing that the C‐terminal region of the gatekeeper MxiC is essential for substrate secretion regulation (Roehrich et al., 2017). Remarkably, the last 11 residues of SepL are also involved in EscD and Tir binding (Wang et al., 2008), suggesting a plausible model for SepL control of substrate secretion that could involve its dynamic interactions with EscD, Tir, and EscV. The interaction of SepL with Tir was proposed to delay effector secretion (since Tir secretion is required for the secretion of other effectors (Thomas, Deng, Baker, Puente, & Finlay, 2007)), while its interaction with EscD was suggested to release Tir and promote effector secretion (Wang et al., 2008). This is supported by the finding that overexpression of *escD* leads to hypersecretion of Tir (Ogino et al., 2006). In addition, since SepL resembles a T3 effector but without being secreted (Younis et al., 2010), it could function as a Trojan horse that, after its recognition as a T3 substrate, blocks a substrate acceptor site on EscV, or modifies the affinity of this export gate component for different substrates. Nonetheless, whether the SepL–EscV interaction participates in secretion regulation remains to be investigated.

**Figure 6:**
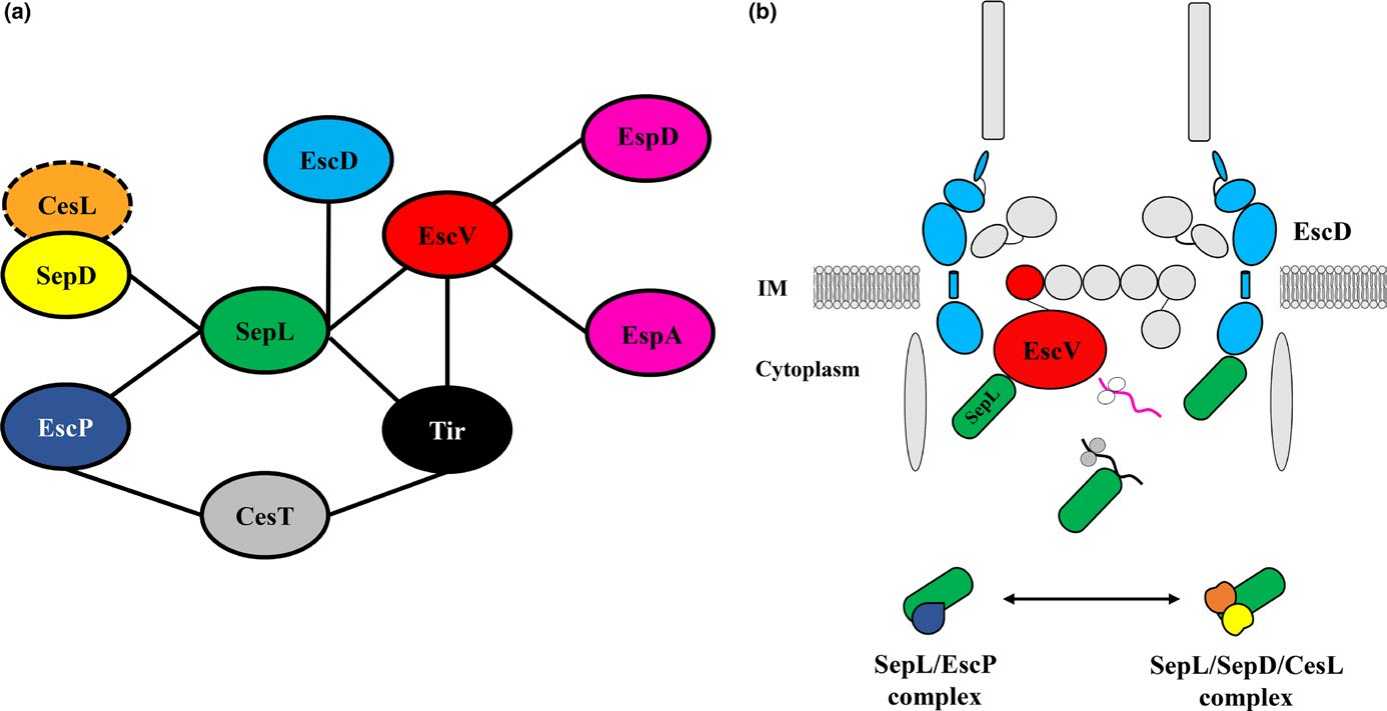
SepL protein–protein interaction network involved in middle and late substrate secretion regulation. **(**a) The SepL protein binds directly to five different T3SS components: SepD, EscP, Tir, EscD, and EscV. Although SepL forms a ternary complex with SepD and CesL (Younis et al., 2010), no direct interaction between CesL (dashed circle) and SepL or SepD has been reported. The effector Tir binds to its cognate chaperone CesT (Elliott et al., 1999), which in turn has been demonstrated to interact with EscP (Monjaras Feria et al., 2012). Finally, the interaction between EscV and different T3S substrates is also displayed. (b) Schematic representation of the EPEC T3SS base showing the SepL binding partners described so far. SepL [green] interacts with EscD [cyan], Tir [black] (Wang et al., 2008), and EscV [red] (this work). In addition, SepL was reported to form two mutually exclusive complexes: the SepL‐EscP [blue] complex, which was proposed to regulate substrate secretion in response to calcium changes in vitro (Shaulov et al., 2017); and the SepL‐SepD [yellow]‐CesL [orange] complex (Younis et al., 2010), whose role in substrate secretion regulation remains to be determined

Furthermore, it was recently shown that SepL interacts with the molecular ruler protein EscP, and that the SepL/EscP complex is stabilized by calcium, preventing effector secretion. The model proposed that once the translocation pore is assembled in the host cell membrane, the calcium flux to the T3SS base is reduced, resulting in dissociation of the SepL/EscP complex and effector secretion (Shaulov et al., 2017). Thus, the EscP switch protein, which has been shown to play a role in effector secretion control (Monjaras Feria et al., 2012), emerges as an important player in the in vitro calcium‐dependent SepL secretion regulatory pathway (Shaulov et al., 2017). Besides, EscP was previously shown to bind to the cognate Tir chaperone CesT, and suggested to act, together with Tir and the SepL/SepD complex, in the restriction of effector secretion (Monjaras Feria et al., 2012). Overall, the intricate interaction network in which SepL participates may reflect the different regulation layers required to fine‐tune the timing of substrate secretion (Figure 6). However, based on previous observations and our results (i.e., neither the translocon senses the absence of calcium nor the SepL/SepD/CesL switch complex is affected by calcium changes), we suggest that the molecular mechanism triggering effector secretion upon in vitro calcium removal could be different from the one triggering effector translocation upon host cell contact, as outlined below.

It is noteworthy that in vitro calcium depletion acts as a common environmental cue that promotes effector secretion in phylogenetically distant bacteria (e.g., *Yersinia*,* Pseudomonas*,* Chlamydia, Vibrio,* and A/E pathogens) (Cisz et al., 2008; Deng et al., 2005; Gode‐Potratz et al., 2010; Ide et al., 2003; Jamison & Hackstadt, 2008; Kim et al., 2005; Michiels, Wattiau, Brasseur, Ruysschaert, & Cornelis, 1990; Sarty et al., 2012; Shaulov et al., 2017), probably by inducing conformational changes in the needle structure that mimic those adopted upon host cell contact (Torruellas et al., 2005). However, by increasing host intracellular calcium levels, Cisz et al. (2008) demonstrated that the in vivo translocation of effectors is not influenced by calcium concentration changes. Therefore, a distinction should be made between induction of effector secretion in vitro and upon host cell contact. While the former can promote effector secretion in a translocator‐independent mechanism (Figure 2) that involves the SepL/EscP protein complex in EPEC (Shaulov et al., 2017), the mechanism participating in effector translocation in vivo is still largely unknown. Finally, our data revealed EscV as a new player that serves as a docking site for both SepL and T3 substrates. Nevertheless, whether EscV acts in concert with the SepL/SepD/CesL regulatory switch complex to ensure the proper timing of substrate secretion remains to be determined.

## CONFLICT OF INTEREST

None declared.
