# Supplementary material for: Novel insights into the mechanism of SepL‐mediated control of effector secretion in enteropathogenic Escherichia coli
Source: Microbiologyopen. 2017 Dec 26;7(3):e00571. doi: 10.1002/mbo3.571 (PMC6011996; doi:10.1002/mbo3.571)
Supplement: Supplementary file 1 [file MBO3-7-e00571-s001.docx]

**
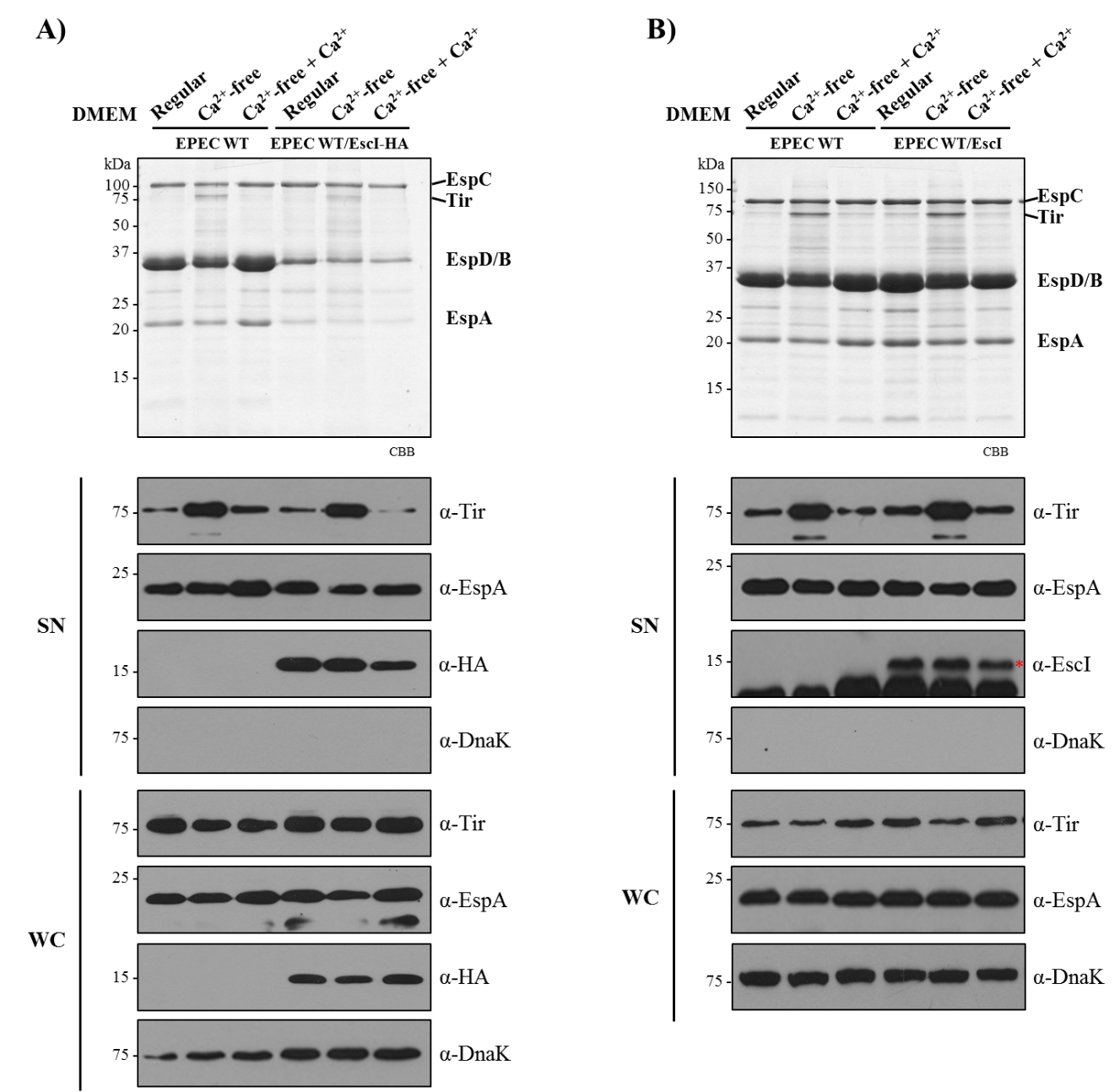
**

**Figure S1. EscI secretion is not influenced by calcium depletion**. A) T3 secretion profiles of EPEC wild-type strain (WT) alone or transformed with EscI-HA grown in regular DMEM containing 1.8 mM of CaCl_2_ (Regular), calcium-free DMEM (Ca^2+^-free) or calcium-free DMEM supplemented with 1.8 mM of CaCl_2_ (Ca^2+^-free + Ca^2+^), visualized by SDS-PAGE stained with Coomassie brilliant blue (CBB) (upper panel). The presence of Tir, EspA, EscI-2HA and DnaK in the supernatants (SN) or whole-cells (WC) was examined by immunoblotting, using anti-Tir, anti-EspA, anti-HA and anti-DnaK antibodies (lower panels). B) Protein secretion profiles of EPEC WT alone or overproducing untagged EscI (EPEC WT/EscI) grown in the presence or absence of calcium as described in A. Immunodetection of Tir, EspA and DnaK was performed in the supernatants (SN) and whole cell lysates (WC) using the specific antibodies anti-Tir, anti-EspA and anti-DnaK (lower panels). The presence of EscI in the supernatants (indicated by a red asterisk) was examined using specific antibodies against the protein (anti-EscI).

**
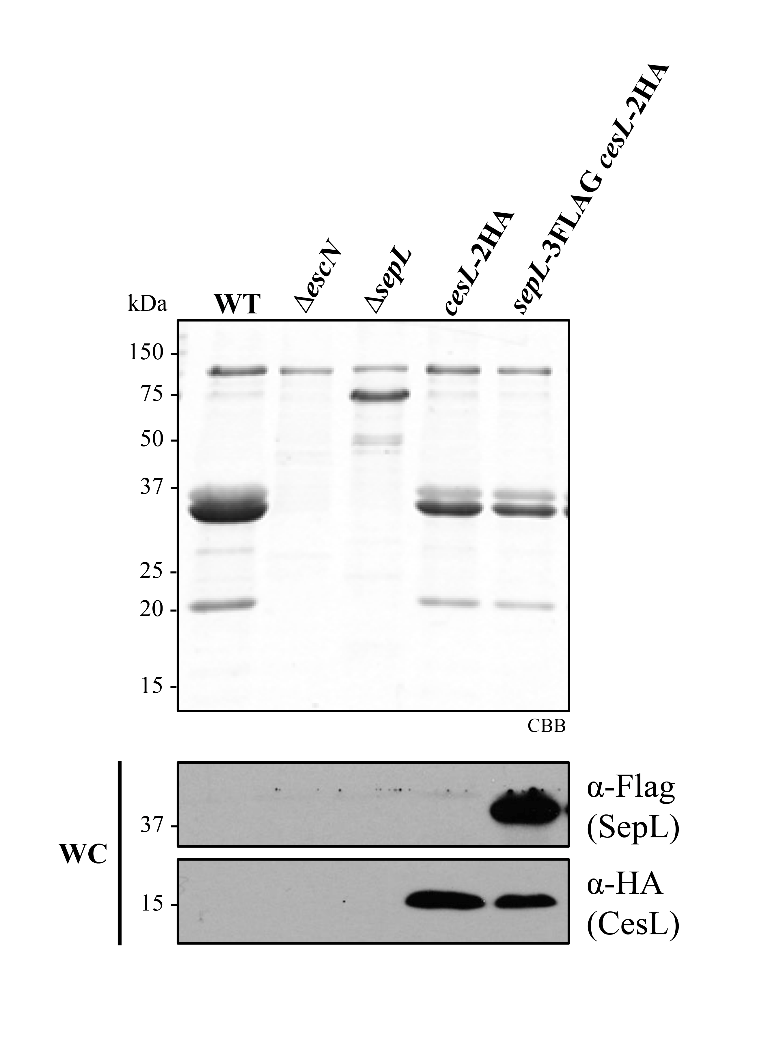
**

**Fig S2. The secretion profile of EPEC *cesL*-2HA and EPEC *sepL*-3FLAG *cesL*-2HA is comparable to that of the wild-type strain.** Protein secretion profiles of EPEC wild-type (WT), ∆*escN*, ∆*sepL*, EPEC *cesL*-2HA and EPEC *sepL*-3FLAG *cesL*-2HA strains. Secreted proteins were visualized by SDS-PAGE stained with Coomassie brilliant blue (upper panel). Production of SepL-3FLAG and CesL-2HA in whole cell lysates (WC) was examined by immunoblotting using anti-FLAG and anti-HA antibodies.

**
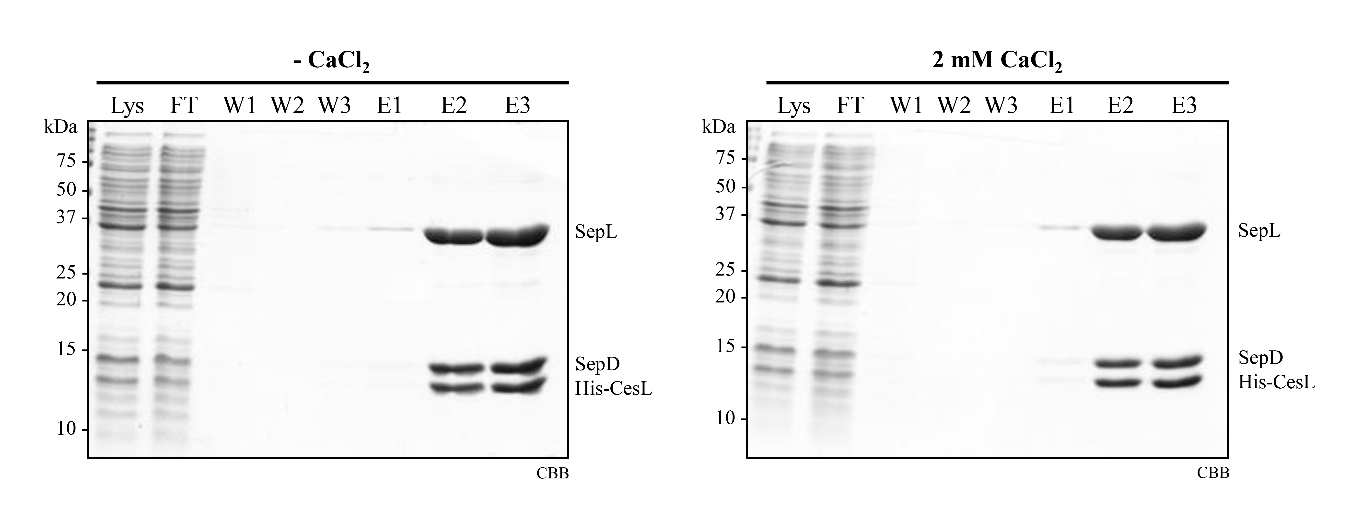
**

**Fig S3. The formation of the SepL/SepD/CesL protein complex occurs in a calcium-independent manner.** Pull-down assays of His-CesL, SepD and SepL performed by nickel affinity chromatography in the absence or presence of 2 mM CaCl_2_. *Salmonella* SJW1368 cells were co-transformed with plasmids pMTBISpDcL and pMATpL for overproduction of SepD and His-CesL, and SepL, respectively. Cleared lysates containing His-CesL, and untagged SepD and SepL (Lys) were incubated with Ni-NTA agarose beads in the absence or presence of 2 mM CaCl_2._ The protein-coupled beads were loaded into a column and the flow through (FT) was collected. After extensive washing (W1, W2 and W3) proteins were eluted (E1, E2 and E3). All samples were visualized by SDS-PAGE stained with Coomassie brilliant blue.

**
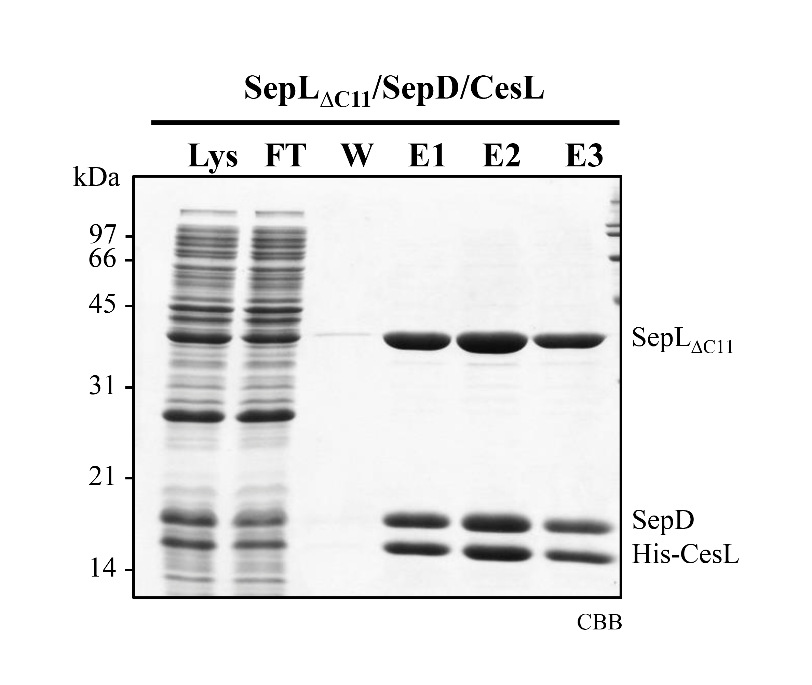
**

**Fig S4. SepL_∆C11_ is able to form a ternary complex with SepD and CesL.** Pull-down assay of His-CesL, SepD and SepL_∆C11_. The cleared lysate containing His-CesL, and untagged SepD and SepL_∆C11_ (Lys) was incubated with Ni-NTA agarose beads_._ The protein-coupled beads were loaded into a column and the flow through (FT) was collected. The column was extensively washed (W) and eluted (E1, E2 and E3). The co-purified proteins were visualized by SDS-PAGE stained with Coomassie brilliant blue.
